# Left Gastric Artery Pseudoaneurysm Due to Pancreatitis

**DOI:** 10.7759/cureus.20405

**Published:** 2021-12-14

**Authors:** Naveen Kumar Gaur, Oseen Shaikh, Sree Subramaniyan S, Abhinaya Reddy, Uday Kumbhar

**Affiliations:** 1 Surgery, Jawaharlal Institute of Postgraduate Medical Education and Research, Puducherry, IND; 2 Surgery, Jawaharlal Institute of Postgraduate Medical Education and Research, Pondicherry, IND

**Keywords:** digital subtraction angiography, embolization, pancreatitis, left gastric artery, pseudoaneurysm

## Abstract

Acute or chronic pancreatitis can cause pseudoaneurysms of visceral arteries. The left gastric artery (LGA) is the least common visceral artery being affected. Here, we report a case of chronic pancreatitis with a pseudoaneurysm of the LGA. A 42-year-old male, a chronic alcoholic, and smoker, presented with abdominal pain, haematemesis, and melena. Diagnosis of pseudoaneurysm of LGA aneurysm was confirmed by computed tomography abdomen. The endovascular coil embolization was done successfully, following which the patient had an uneventful recovery.

## Introduction

Pancreatitis is a common condition and can occur following various etiologies. Pancreatitis is associated with a pseudocyst, pseudoaneurysm, gastric outlet obstruction, and splenic vein thrombosis. Complications like pseudoaneurysm are rare. Patients with pseudoaneurysm may be asymptomatic or present as an emergency with hematemesis and melena. Rarely this aneurysm may present as spontaneous intraperitoneal bleed [1]. Splenic artery pseudoaneurysm is most common following pancreatitis [2]. Pseudoaneurysm of the left gastric artery (LGA) is rare and accounts for less than 4% of the total cases [3,4]. Early diagnosis with essential radiological investigations is most important for these patients as they can lead to dangerous complications like upper gastrointestinal bleed or spontaneous intraperitoneal bleed. Early diagnosis reduces mortality and morbidity. We report an LGA pseudoaneurysm case, which was successfully managed with trans-arterial embolization.

## Case presentation

A 42-year-old male, a chronic alcoholic, and smoker presented to the emergency department with complaints of hematemesis and melena for one day. He had a history of pain abdomen for the past three months aggravated for the past 10 days. The pain was dull, aching localized to the epigastric region with radiation to the back. He had no history of jaundice, reduced urine output, breathlessness, giddiness. The patient had multiple episodes of abdominal pain and was diagnosed to have pancreatitis one year ago. On examination, he was conscious and oriented. His pulse rate was 98 beats per minute, and his blood pressure was 100/60 mmHg. On examination, epigastric tenderness was present, and the rest of the abdomen was soft. There was no guarding or rigidity, and bowel sounds were normal.

Blood examination showed hemoglobin of 8.8 g/dl, with normal total leukocyte and platelet counts. Renal parameters and serum electrolytes were normal. Liver function tests were normal. His amylase was 556 IU/L. Upper gastrointestinal endoscopy showed altered blood in the stomach without any active bleeding.

**Figure 1:**
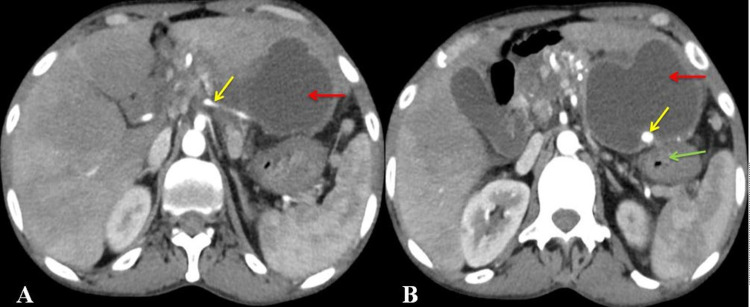
Contrast-enhanced computed tomography of the abdomen (axial view) showing: A: Origin of the left gastric artery (yellow arrow) and acute fluid collection (red arrow), and B: pseudoaneurysm of the left gastric artery (yellow arrow), acute fluid collection (red arrow), and stomach (green arrow).

An ultrasonogram (USG) of the abdomen showed an anechoic collection of size 5 cm x 8 cm x 3 cm with subtle moving echoes in the lesser sac, posterior to the stomach. Another anechoic collection measured 8 cm x 5 cm x 4 cm posterior to segment IV of the liver. However, the pancreas could not be visualized. Contrast-enhanced computed tomography (CT) abdomen showed calcification in the pancreas with surrounding fat stranding. The two large collections measured 10 cm x 10 cm x 6 cm and 10 cm x 6 cm x 5 cm in the lesser sac and left lobe subcapsular region, respectively. There was the presence of the LGA pseudoaneurysm measuring 8 mm x 6 mm x 6 mm within and at the posteroinferior aspect of the first collection, without active contrast extravasation (Figure 1).

**Figure 2:**
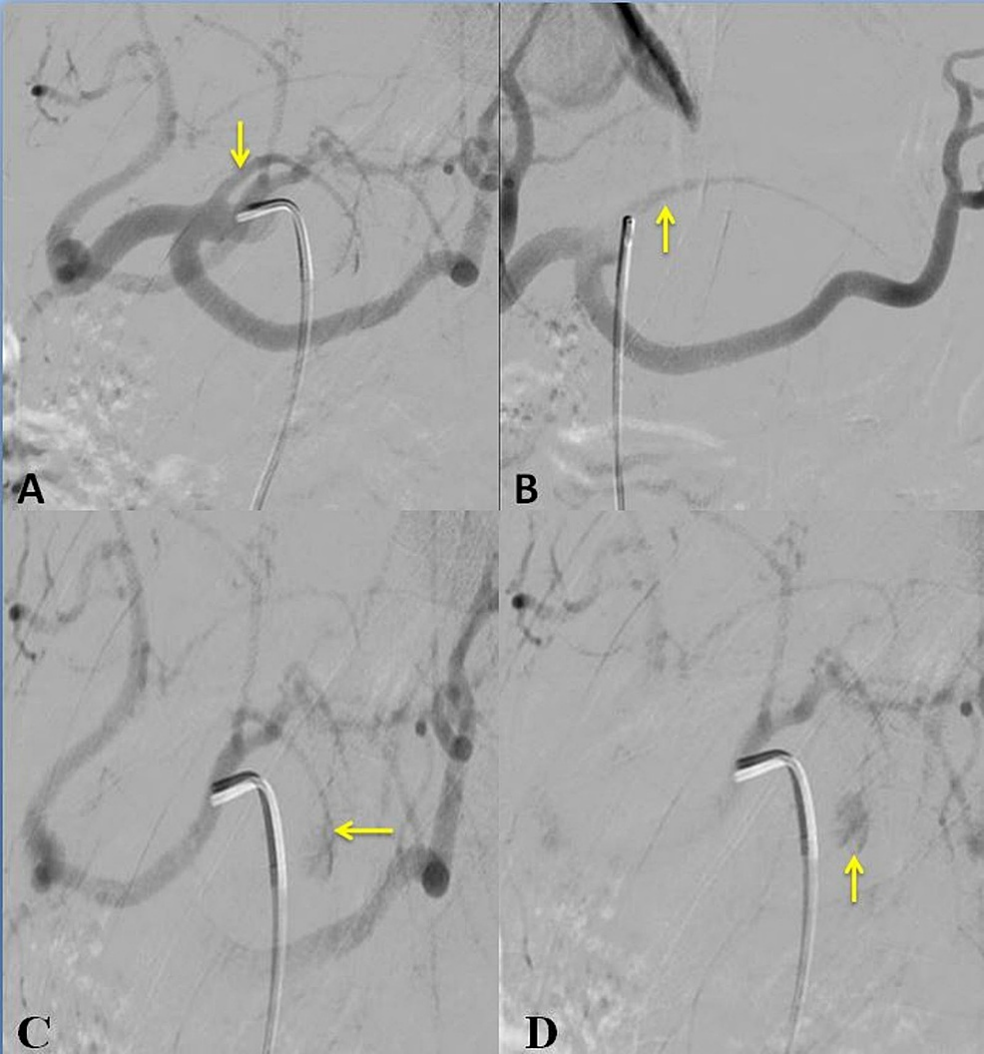
Digital subtraction angiography showing: A: Left gastric artery origin (yellow arrow); B: course of the left gastric artery (yellow arrow); C and D: pseudoaneurysm of the left gastric artery (yellow arrow).

The patient underwent digital subtraction angiography (DSA) using a 5 Fr diagnostic catheter. The celiac trunk was catheterized. DSA showed a slow filling pseudoaneurysm arising from the left gastric artery (Figure 2).

**Figure 3:**
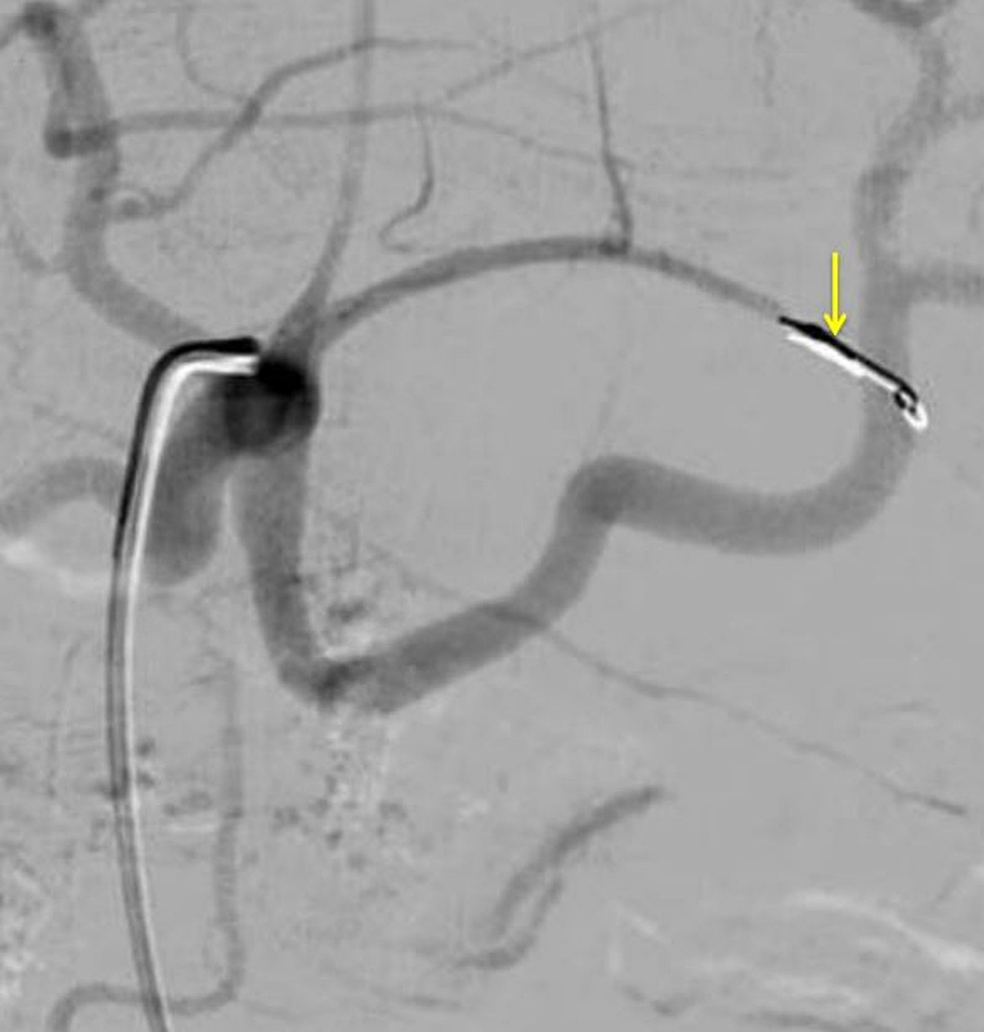
Digital subtraction angiography showing coil embolization of the left gastric artery (arrow).

Our patient was diagnosed to have an LGA pseudoaneurysm. The patient was planned for trans-arterial embolization. The LGA was super selectively catheterized using a Progreat microcatheter and embolized the pseudoaneurysm using a pushable coil (Figure 3).

**Figure 4:**
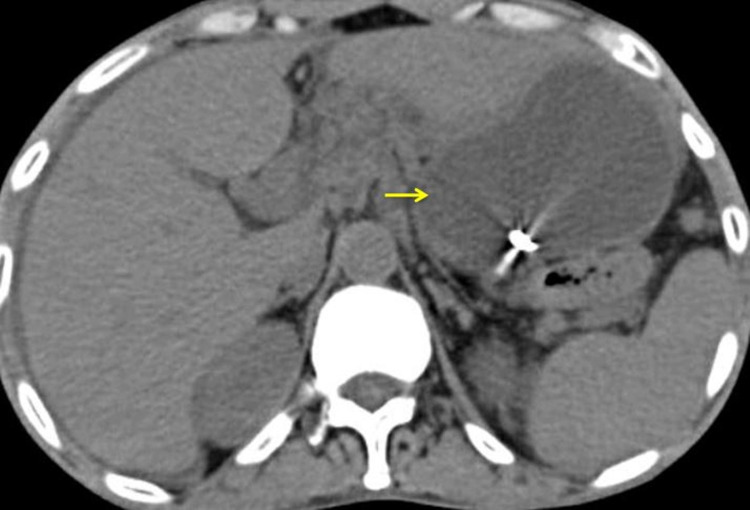
Computed tomography (axial view) done after the embolization showing coil in the left gastric artery.

Post embolization check DSA showed no leak or extravasation, indicating complete embolization of the LGA pseudoaneurysm. A check CT scan of the abdomen was done post-procedure, which showed complete thrombosis of the aneurysm (Figure 4).

He was discharged and was advised for regular follow-up. The patient was being followed up after one month and six months. There was no recurrence of gastrointestinal bleed.

## Discussion

Pancreatic pseudoaneurysms, although an uncommon clinical entity, pose a severe risk of untoward complications [2,5]. Pancreatic pseudoaneurysms can occur as pancreatitis or following pancreatic surgeries, the former being more common. The splenic artery is the most common visceral artery affected for the formation of pseudoaneurysm as a result of pancreatitis [6]. Other vessels which can be affected are gastroduodenal, pancreaticoduodenal, hepatic, and rarely left gastric arteries. Pseudoaneurysms of the LGA are rare, and very few cases have been reported until now. LGA arises from the celiac axis, divides into small branches, and supply anterior and posterior gastric surfaces. One of these branches turns sharply downward and travels along the lesser curvature to anastomose with the right gastric artery branches. They can arise in the portion of the artery lying outside the gastric wall (extramural) or within the gastric wall (intramural); both types, if ruptured, may result in severe life-threatening complications due to intra-gastric or intra-peritoneal spontaneous massive hemorrhage [7]. Pseudoaneurysm of the splenic artery is more common than LGA. It may be because the splenic artery is relatively fixed retroperitoneally and in contact with the pancreas throughout its course, compared to the LGA, which is more mobile due to the peritoneal covering. In our patient, involvement of the LGA was probably because the LGA was traversing through the collection.

Pseudoaneurysm develops due to autodigestion of the arterial wall by the pancreatic enzymes like amylase, lipase, and protease or an anastomotic leak after pancreatic surgeries, which is rich in pancreatic enzymes. Sooner or later, lysis of tunica intima and media will occur, leading to focal dilation of the vessel and the formation of pseudoaneurysm [5,7].

Pseudoaneurysm may present as hematemesis or melena if it ruptures into the gastrointestinal tract. It may present as hemosuccus pancreatitis if it ruptures into the pancreatic duct or indirectly via pseudocyst. It can also present as an intraperitoneal or retroperitoneal hemorrhage [8,9]. The mortality rate is as high as 90% for ruptured pseudoaneurysms. Although the clinical picture of LGA pseudoaneurysm may not be different from the other visceral artery pseudoaneurysm, treating surgeons should remember that even LGA pseudoaneurysm is also possible. Our patient had presented with features of pancreatitis and upper gastrointestinal bleed.

USG of the abdomen may show changes of acute or chronic pancreatitis. We may not be able to see the aneurysm unless very large. However, it has the disadvantage of inter-observer variations. Doppler ultrasound is the practical and preferred choice for screening small pseudoaneurysms. Usually, they appear as a hypoechoic cystic structure adjacent to the supplying artery. Doppler ultrasound may show a characteristic “ying-yang sign.” The to-and-fro movement of the blood from the neck to the aneurismal sac produces this sign [10]. Contrast-enhanced CT provides the site, size, and extent of pseudoaneurysm of the involved artery and its adjacent effect on viscera [7]. Risk stratification can be done in pancreatitis using a modified CT severity index score to anticipate unexpected outcomes and follow up with further necessary investigations [11]. In contrast-enhanced CT, using volume rendering, multi-planar techniques, three-dimensional reconstruction can be done, which can be helpful in pre-procedural planning and individualizing the anatomy of arterial structures in the celiac axis.

Magnetic resonance angiography (MRA) can also be used with the advantage of lacking radiation exposure but has a minimal role in emergencies [4]. DSA is the gold standard armamentarium of choice in diagnosis, management, and follow-up. DSA can also provide accurate details of the flow, site, and size of the pseudoaneurysm [12]. In addition, recent literature suggests that endoscopic ultrasound with doppler functionality can be a useful tool in diagnosing gastric pseudoaneurysms [10]. Intramural type of gastric aneurysms can present as submucosal tumor-like swelling during endoscopy. In such cases, endoscopic ultrasound (EUS) with added doppler function can help study the gastric wall in detail and diagnose submucosal pseudoaneurysms [10]. In our patient, we did contrast-enhanced CT abdomen and DSA, which were diagnostic of LGA aneurysm.

Treatment of patients with LGA pseudoaneurysm is by endovascular approach or by surgery. Endovascular management includes super-selective catheterization of the involved artery and trans-arterial embolization using particle agents, liquid agents, or mechanical agents, which can be used alone or in combination [13]. Moreover, early embolization can help reduce the number of transfusions to be given. DSA has a very low incidence of postoperative complications, morbidity, and mortality [14]. If such facilities are unavailable or endovascular management has failed, or the patient is unstable due to rupture of an aneurysm, then surgery is the better option. Surgical options include resection and bypass procedure, arterial ligation, organ removal [7]. Our patient was treated with an endovascular approach, and trans-arterial embolization was done using coils, as the patient was stable throughout the hospital.

## Conclusions

LGA pseudoaneurysms are very rare, and few cases are reported in the literature. The treating physician should be aware of such a rare presentation. High clinical suspicion should be there in patients of chronic pancreatitis with signs of bleeding and unstable vitals. Trans-arterial embolization is a primary management option if the patient is stable.
